# DASS-9: Hacia una medición más eficiente de síntomas emocionales

**DOI:** 10.15446/rsap.V27n2.114528

**Published:** 2025-03-01

**Authors:** Carlos Iván Orellana, Ligia María Orellana

**Affiliations:** 1 CO: Psicól. Ph. D. Ciencias Sociales. Departamento de Psicología y Salud Pública, Universidad Centroamericana José Simeón Cañas. La Libertad, El Salvador. corellana@uca.edu.sv Universidad Centroamericana José Simeón Cañas Departamento de Psicología y Salud Pública Universidad Centroamericana José Simeón Cañas La Libertad El Salvador corellana@uca.edu.sv; 2 LO: Psicól. Ph. D. Psicología. Departamento de Psicología, Universidad de La Frontera. Temuco, Chile. ligia.orellana@ufrontera.cl Universidad de La Frontera Departamento de Psicología Universidad de La Frontera Temuco Chile ligia.orellana@ufrontera.cl

**Keywords:** Psicometría, depresión, ansiedad, estrés, prevalencia, incidencia, salud mental *(fuente: DeCS, BIREME)*, psychometrics, depression, anxiety, stress, prevalence, incidence, mental health *(source: MeSH, NLM)*

## Abstract

**Objetivos:**

Exponer las características métricas y las bondades prácticas de la DASS-9, una versión reducida de la conocida escala DASS-21 para medir síntomas emocionales.

**Métodos:**

A partir de datos de una investigación previa realizada con población adulta salvadoreña se elaboró una escala de nueve ítems para medir sintomatología emocional.

**Resultados:**

La DASS-9 presenta la estructura factorial tríadica esperada (depresión, ansiedad y estrés), altos niveles de consistencia interna (a > 0,86) e indicios de validez de criterio-concurrente (i. e., correlaciones con factores de disrupción cotidiana o miedo) que emulan los resultados de la investigación de origen, pero con una reducción de casi el 60% de los ítems originales.

**Conclusiones:**

La DASS-9 presenta propiedades psicométricas adecuadas y constituye una alternativa más eficiente para el estudio de síntomas emocionales en población salvadoreña y latinoamericana. La amplia utilización de la escala DASS-21 en Latinoamérica durante la pandemia y la actual alta incidencia de problemas emocionales a nivel global, vuelven aconsejable crear escalas más breves que fortalezcan el interés y la investigación sobre sintomatología emocional.

## Abstract (en)

**Objectives:**

To examine the metric properties and practical benefits of the DASS-9, a shortened version of the well-known DASS-21, a scale for measuring emotional symptoms.

**Methods:**

Based on data from previous research with Salvadoran adult population, a 9-item scale was developed to measure emotional symptomatology.

**Results:**

The DASS-9 shows the expected triadic factorial structure (depression, anxiety, and stress), high levels of internal consistency (a > 0.86) and evidence of criterion-concurrent validity (i.e., correlations with daily disruption or fear of contagion) that emulate the results of the original research, but with a cutoff of almost 60% of the original items.

**Conclusions:**

The DASS-9 shows adequate psychometric properties and is a more efficient alternative for the study of emotional symptoms in Salvadoran population, and in Latin American population as well. The wide use of the DASS-21 in Latin America during the pandemic and the current high incidence of emotional problems globally, make it advisable to create shorter scales that strengthen interest and research on emotional symptomatology.

El constante interés científico y de salud pública por los síntomas emocionales comórbidos y comunes, como la depresión o la ansiedad, se vio intensificado durante la pandemia de COVID-19. Este interés persiste, a pesar de que la alerta sanitaria fue suspendida, debido a la alta incidencia y el impacto humano de estos síntomas 1-5.

Según el Programa de las Naciones Unidas para el Desarrollo (PNUD) 6, la pandemia exacerbó tendencias preexistentes de angustia, y en la actualidad hasta tres billones de personas experimentarían cotidianamente sentimientos de preocupación, estrés y tristeza. No obstante, aunque se constata la presencia, el impacto y el pronóstico deletéreo de estos síntomas en países específicos 1,7,8, persiste el desconocimiento sobre las secuelas de la pandemia, especialmente las de carácter emocional 9,10. Asimismo, más allá de la pandemia como circunstancia extraordinaria y exacerbante de problemas colectivos, y tomando distancia de explicaciones psicologistas o biologistas, en Latinoamérica hay que sumar condiciones contextuales adversas inamovibles (e. g., desempleo, inseguridad ciudadana) o en franco deterioro (i. e., cambio climático, autoritarismo, desigualdad) que explicarían mejor por qué la prevalencia de depresión que experimenta esta región (12%) es más del doble en comparación con la que se registra mundialmente (5%) 11.

Ante este panorama, se vuelve necesario contar con instrumentos adecuados para proseguir e incentivar la exploración de síntomas emocionales como la depresión, la ansiedad y el estrés en países latinoamericanos. El objetivo de este escrito es instrumental y consiste en exponer las características psicométricas de una escala abreviada para medir síntomas emocionales: el DASS-9.

## MÉTODOS

Los pormenores metodológicos del estudio de base -muestra, instrumentos y procedimiento- pueden ser consultados en la investigación original 8. En esta, 339 personas adultas salvadoreñas contestaron un cuestionario que incluyó la conocida escala abreviada de Depresión, Ansiedad y Estrés, DASS-21.

El instrumento de 21 ítems mostró adecuadas propiedades psicométricas: estructura factorial de tres factores de siete ítems cada uno; consistencia interna (alfa de Cronbach) satisfactoria para la escala total (0,95) y para cada uno de los tres factores (0,87 en promedio); también, indicios de validez de criterio-concurrente al encontrar niveles estadísticamente mayores de síntomas emocionales en mujeres que en hombres, similar a resultados encontrados a nivel mundial 3, además de correlaciones positivas de sus dimensiones con indicadores de perjuicio situacional, como miedo al contagio y percepción de deterioro de relaciones en el hogar 8,12.

Para obtener una escala muy breve para medir síntomas emocionales a partir de la DASS-21 aplicada en la investigación original, se procedió de la siguiente manera: de cada subescala de síntomas emocionales -depresión, ansiedad y estrés-, cada una originalmente compuesta por siete ítems, se seleccionaron aquellos tres con las cargas intrafactoriales más altas 12. Esto redujo la escala total original, compuesta por 21 ítems, a un instrumento conformado por las mismas tres subdimensiones de interés, pero ahora cada una compuesta solo por tres ítems, para totalizar nueve ítems (una reducción de casi el 60% de los ítems de la DASS-21). Cabe apuntar que, en países fuera de Latinoamérica, existen iniciativas similares de reducción del DASS-21 y todos los ítems de estas escalas reducidas 13,14 se encuentran presentes en el DASS-9.

## RESULTADOS

La Tabla 1 muestra las evidencias psicométricas principales del ejercicio de reducción de la escala DASS-21 para obtener la escala DASS-9. Como puede apreciarse, la nueva escala reducida presenta validez de constructo al replicar la estructura tridimensional del DASS-21. Asimismo, cada factor presenta altos niveles de consistencia interna, al igual que la escala total (a = 0,93). Por otra parte, es posible añadir que la nueva escala y sus sub-dimensiones replican los indicios de validez-concurrente del estudio original. Por ejemplo, los niveles de síntomas emocionales medidos por la escala total [t (337) = 2,084, p<0,038)] son estadísticamente mayores en mujeres que en hombres (respectivamente, M = 8,0, DE = 6,8 vs. M = 6,4, DE = 5,8). También, las tres subdimensiones correlacionaron fuerte y positivamente entre sí (r promedio = 0,77, p<0,001), al igual que cada dimensión lo hace con indicadores como la percepción de deterioro de las relaciones en el hogar (r promedio = 0,34, p<0,001) o con el miedo al contagio (r promedio = 0,24, p<0,001).

**Tabla 1:** Ítems, alfa de Cronbach, cargas factoriales y varianzas de las subescalas del DASS-9

Ítems	F1	F2	F3
F1: Ansiedad (α = 0,86)			
15 - Estuve a punto de sentir pánico	0,677		
19 - Sentí los latidos de mi corazón, a pesar de no haber hecho ningún esfuerzo físico	0,862		
20 - Tuve miedo sin razón	0,785		
F2: Depresión (α = 0,88)			
10 - He sentido que no había nada que me ilusionara		0,856	
13 - Me sentí triste y deprimido		0,675	
16 - No me pude entusiasmar por nada		0,811	
F3: Estrés (α = 0,86)			
1- Me ha costado mucho descargar la tensión			0,824
11- Me he sentido inquieto			0,728
12 - Se me hizo difícil relajarme			0,732
Autovalor	5,728	0,766	0,670
Porcentaje de varianza explicada posterior a la rotación	27,222	26,277	26,102
Porcentaje de varianza acumulada posterior a la rotación	27,222	53,498	79,601

F= Factor. Los números de cada ítem corresponden a la numeración original del DASS-21 15. El análisis factorial confirmatorio (AFC) recurrió a una rotación Varimax y su ejecución comprobó el cumplimiento de los supuestos para llevar a cabo el análisis (KMO = 0,92; P. esfericidad de Bartlett: p<0,001).

## DISCUSIÓN

La alta incidencia actual y los efectos deletéreos, por venir de síntomas emocionales como la depresión, la ansiedad y el estrés 4,6, requerirán el refinamiento de las herramientas de investigación disponibles. Aunque la escala DASS-21 ha mostrado ser efectiva para la medición de estos síntomas en distintos países de Latinoamérica - incluyendo El Salvador como contexto de referencia de este escrito 8,12,16-, otros trabajos realizados fuera de Latinoamérica 13,14 demuestran la necesidad de contar con escalas de medida psicométricamente sólidas, pero también más concisas y eficientes.

La justificación del acortamiento de la DASS-21 es triple. Primero, en términos técnicos, la DASS-21 parece seguir acusando el problema por el cual, en su momento, se redujo la escala original DASS-42, esto es, su invariable alta consistencia interna, fruto de la redundancia de sentido entre los ítems de las distintas dimensiones. En segundo lugar, una razón teórico-fenomenológica, pues la depresión, la ansiedad y el estrés, aunque constituyen afecciones comórbidas, tienen expresiones bastante distintivas que permiten mediciones alternas largas o muy breves 17. Por último, en términos pragmáticos, su uso generalizado y contextualizado en aras de estimular la investigación. Muchos investigadores podrían beneficiarse del uso de la DASS-9 que aquí se expone o, mejor aún, podrían ensayar la creación de escalas recortadas propias partir de los datos contextualmente respaldados con los que cuentan al haber administrado en alguna ocasión la DASS-21.

El carácter consuetudinario, comórbido y la alta incidencia de los síntomas emocionales 4,5, la indefinida situación pospandemia y su plétora de efectos 2,7, sumado a las siempre inestables condiciones sociales, políticas, económicas y ambientales de Latinoamérica 11, como caldo de cultivo contextual atentatorio contra la salud mental, convierten el estudio de los síntomas emocionales en un imperativo científico que requiere contar con las mejores herramientas posibles.

Dadas las propiedades psicométricas adecuadas de la DASS-9 aquí expuestas, y que hasta la fecha se desconoce la existencia de propuestas de reducción del DASS-21 en países de habla hispana, se concluye que la DASS-9 se convierte en una sugerencia técnica viable para la comunidad de personas investigadoras. Asimismo, este instrumento constituye una alternativa apropiada para el diagnóstico o la investigación -clínica, social o de salud pública- sobre síntomas emocionales en países latinoamericanos.
